# Predicting dental anxiety in young adults: classical statistical modelling approach versus machine learning approach

**DOI:** 10.1186/s12903-024-04012-3

**Published:** 2024-03-09

**Authors:** Chukwuebuka Ogwo, Wisdom Osisioma, David Ifeanyi Okoye, Jay Patel

**Affiliations:** 1 Department of Oral Health Sciences, Maurice H Kornberg School of Dentistry, Temple University, Philadelphia, PA 19140 US; 2 School of Public Health, Texas A&M University, College Station, TX USA

## Abstract

**Objectives:**

To predict and identify the key demographic and clinical exposure factors associated with dental anxiety among young adults, and to compare if the traditional statistical modelling approach provides similar results to the machine learning (ML) approach in predicting factors for dental anxiety.

**Methods:**

A cross-sectional study of Western Illinois University students. Three survey instruments (sociodemographic questionnaire, modified dental anxiety scale (MDAS), and dental concerns assessment tool (DCA)) were distributed via email to the students using survey monkey. The dependent variable was the mean MDAS scores, while the independent variables were the sociodemographic and dental concern assessment variables. Multivariable analysis was done by comparing the classical statistical model and the machine learning model. The classical statistical modelling technique was conducted using the multiple linear regression analysis and the final model was selected based on Akaike information Criteria (AIC) using the backward stepwise technique while the machine learining modelling was performed by comparing two ML models: LASSO regression and extreme gradient boosting machine (XGBOOST) under 5-fold cross-validation using the resampling technique. All statistical analyses were performed using R version 4.1.3.

**Results:**

The mean MDAS was 13.73 ± 5.51. After careful consideration of all possible fitted models and their interaction terms the classical statistical approach yielded a parsimonious model with 13 predictor variables with Akaike Information Criteria (AIC) of 2376.4. For the ML approach, the Lasso regression model was the best-performing model with a mean RMSE of 0.617, R^2^ of 0.615, and MAE of 0.483. Comparing the variable selection of ML versus the classical statistical model, both model types identified 12 similar variables (out of 13) as the most important predictors of dental anxiety in this study population.

**Conclusion:**

There is a high burden of dental anxiety within this study population. This study contributes to reducing the knowledge gap about the impact of clinical exposure variables on dental anxiety and the role of machine learningin the prediction of dental anxiety. The predictor variables identified can be used to inform public health interventions that are geared towards eliminating the individual clinical exposure triggers of dental anxiety are recommended.

**Supplementary Information:**

The online version contains supplementary material available at 10.1186/s12903-024-04012-3.

## Introduction

Dental fear or anxiety (DA) can be described as a subjective negative reaction to dental treatment resulting from a learned negative behavior and often attributed to the aggressive conditioning process which occurred during childhood [1]. Fear of pain has been found to be the main cause of anxiety and a major barrier to seeking dental care [2, 3]. Dental avoidance has been linked with dental fear and anxiety in many patients and thus has led to the deterioration of their oral health state [4, 5]. In severe cases of dental anxiety, the dentist-patient relation may be hampered and sometimes lead to misdiagnoses of anxiety for pain which might result in wrong treatment administration [4, 6]. The standardized and validated tool for the measurement of dental anxiety is known as the Modified Dental Anxiety Scale (MDAS) [7].

Globally, several reports have shown the prevalence of dental anxiety to be between 2.5 and 20% depending on population and methods of assessment [8–10]. Studies have also reported dental anxiety to occur more in females than males [7, 11]. Recent studies have shown that 51% of subjects reported dental anxiety onset in childhood, 22% in adolescence, and 27% in adulthood [8, 12]. Studies have shown gender and age differences in the prevalence of dental anxiety but more importantly, the socioeconomic differences which bother mostly on fear of treatment cost [7, 11, 13].

There are limited studies on the burden of dental anxiety among university students in the US, which constitute a reasonable population of adolescents and young adults in the country [14]. Also, very few studies have explored the clinical exposure variables that may be associated with dental anxiety among young adults [15]. A study of Washington University students showed that about one out of five students reported high levels of dental anxiety (mean DAS = 9.2; SD = 3.4) and most of the students reported that their dental anxiety was due to dental injection [16]. Previous studies in the U.S. have focused on dental anxiety among American children and older adults with only a few studies on young adults or adolescents [14, 15]. Thus, there is a need for more studies among young adults to understand the clinical exposure variables contributing to dental anxiety.

Supervised machine learning (ML) is a subset of artificial intelligence used in the prediction of outcome measures based on several input measures. The goal of the ML model is to optimize the bias-variance trade-off and prevent model underfitting or overfitting [17]. Machine Learningprovides a robust approach for the identification and selection of the most important predictors, without running into issues of numerical convergence and the “curse of dimensionality” a phenomenon common in classical statistical modeling with a lot of predictor variables. Most importantly, there is a dearth of literature comparing classical statistical modeling and ML model in predicting oral health outcomes and more specifically dental anxiety.

The objectives of this study were to predict and identify the key demographic and clinical exposure factors associated with dental anxiety among young adults, and to compare if the traditional statistical modelling approach provides similar results to the machine learning (ML) approach in predicting factors for dental anxiety.

## Methods

This is a cross-sectional study of university students recruited from Western Illinois University (WIU). Eligible participants were graduate and undergraduate students registered for the 2017 Spring semester at WIU at both campuses (Macomb and the Quad Cities campuses). Using the pwr package [18] in R for generalized linear regression, we estimated the sample size of 1062 students based on the following assumptions – small effect size of 2%, 21 variables, and 80% power, and 95% confidence level.

### Data collection

After obtaining approval from the IRB, we obtained the list of emails of the students registered for the Spring semester of 2017 and emailed the survey instruments to the participants electronically via Survey Monkey. The survey instrument consisted of a self-administered structured questionnaire, a validated modified dental anxiety scale (MDAS) [7], and a Dental Concern/Fear Assessment tool (DCA) [19]. The inclusion criteria were all students (undergraduate, graduate, and doctoral) registered for the spring 2017 semester. The e-mails were sent two times, the first one in March and the second one in April 2017. The termination date for the survey return was set at two weeks after the first survey was sent. The survey emails were sent in the evening, assuming that students would be more relaxed in the evenings and have more time to complete the survey. Informed consent was obtained from all subjects and their legal guardian(s) before responding to the questionnaires.

The dependent variable was the mean MDAS scores. The independent variables (see Appendix I for details) include age group, sex, socioeconomic status, dental visit, frequency of dental visit, level of education, and the dental concern variables (Sound or vibration of the drill, dislike of the numb feeling, injection in the mouth, sound or feel of scraping during teeth cleaning, cold air on the teeth, root canal treatment, tooth removal, fear of being injured, panic attacks, fear of feeling pain during treatment, the concern of being embarrassed, smells in the dental office, worried about need a lot of dental treatment, cost of the dental treatment). Studies have identified the cognitive conditioning pathway as a framework elucidating the aetiology of dental anxiety. Within this framework, individuals with negative experiences during dental visits may establish a conditioned association linking the dentist with anxiety [20, 21]. The DCA tool was modified and adapted for our study to consist of 15 short dental anxiety-specific questions with three rank-order response options and was used to measure dental anxiety concerning individual triggers or aggravating factors from dental procedures. Before the primary data collection, a pilot study was conducted using a randomly selected thirty (30) participants to check the response rate, acceptability and the validity of the assessment tools that were utilized for the study.

### Statistical analysis

Only participants who have complete data for all the variables were included in the final analysis. No imputation of data was done. The univariate analyses were conducted for both the dependent and independent variables. The one-way ANOVA test was used to assess the bivariate relationship between each categorical independent variable and the continuous dependent variable (MDAS score). Only the statistically significant variables (*p* < 0.05) were included in the final model for both the classical statistical approach and the ML approach.

Multivariable analysis was done to ascertain the relationship between the independent variables and the MDAS score and identify the predictors of dental anxiety by comparing the results from two modelling approaches: The classical statistical approach and the machine learning approach. The classical statistical modelling technique was conducted using the multiple linear regression analysis and the final model was selected based on Akaike information Criteria (AIC) using the backward stepwise technique. The root mean square error (RMSE), and coefficient of determination (R^2^) for the classical statistical modelling approach was calculated. For comparison, the machine learning technique was performed by comparing two ML models: LASSO regression [22] and extreme gradient boosting machine (XGBOOST) [23] under 5-fold cross-validation using the resampling technique. The data pre-processing (Standardization and normalization) for the machine learning model was done using the recipes package [24]. The model performance was based on RMSE, R^2^, and mean absolute error (MAE). The RMSE value was the main metric for performance assessment and comparison of both model types. The RMSE value indicates measures difference between the predicted values and the observed values. Therefore, the lower the RMSE, the better the model performance. All statistical analyses were performed using R version 4.1.3.

## Results

A total of 454 students (45% response rate) completed the dental anxiety questionnaire (i.e. completely answered at least the Sociodemographic and MDAS sections of the questionnaire) by the end of the survey period and thus were included in the study. No data were inputted. The pilot study of 30 students showed a response rate of 100%. All the 30 participants accepted the questionnaires and had no worries or questions about the content of measurement tools. Thus, the questionnaires were validated for the measurement of dental anxiety within this population and their responses were included in the study.

As shown in Table 1, about two-thirds of the participants were female (69.40%). Most of the respondents were within the age range of 15 to 24 (68.10%). Most of the respondents (98.20%) have visited a dentist. About 35.20% of the respondents had not visited the dentist in the last 12 months. More than half of the respondents reported a household income of $74,999 or less.

**Table 1:** Descriptive statistics of the dental anxiety scores, demographic and dental concern assessment variables

				N		Frequency (%)
Sex		Female		135		69.40
		Male		139		30.60
Age		Less than 24		309		68.10
		25 to 34		84		18.50
		35 to 44		29		6.40
		45 to 54		24		5.30
		55 or older		8		1.80
Dental visit		Yes		446		98.20
		No		8		1.80
Frequency of dental visits^*^		Less than 3 months		115		25.30
		3 months to < 6 months		89		19.60
		6 months to < 12 months		90		19.80
		More than 12 months		160		35.20
Household Income^*^		$0 - $24,999		126		27.80
		$25,000 - $74,999		172		37.90
		$75,000 - $124,999		119		26.20
		$125,000 - $149,999		19		4.20
		$150,000 and up		18		4.00
Level of Education		Doctoral Student		9		2.00
		Graduate Student		142		31.30
		Undergraduate Student		303		66.70
Sound or vibration of the drill		Low		142		31.30
		Moderate		156		34.40
		High		156		34.40
Dislike the numb feeling		Low		262		57.70
		Moderate		152		26.70
		High		71		15.60
Injection in the mouth		Low		97		21.40
		Moderate		152		33.50
		High		205		45.20
The sound or feel of scraping during teeth cleaning		Low		185		40.70
		Moderate		140		30.80
		High		129		28.40
Cold air on the teeth		Low		200		44.10
		Moderate		154		33.90
		High		100		22.00
Root canal treatment		Low		49		10.80
		Moderate		97		21.40
		High		308		67.80
Tooth removal		Low		53		11.70
		Moderate		95		20.90
		High		306		67.40
Fear of being injured		Low		167		36.80
		Moderate		131		28.90
		High		156		34.40
Panic attacks		Low		281		61.90
		Moderate		100		22.00
		High		73		16.10
Fear of feeling pain during treatment		Low		62		13.70
		Moderate		147		32.40
		High		245		54.00
Concern of being embarrassed		Low		269		59.30
		Moderate		102		22.50
		High		83		18.30
Smells in the dental office		Low		327		72.00
		Moderate		89		19.60
		High		38		8.40
Worried about needing a lot of dental treatment		Low		250		55.10
		Moderate		102		22.50
		High		102		22.50
Cost of the dental treatment		Low		127		28.00
		Moderate		140		30.80
		High		187		41.20
**Dental anxiety Level**		**MDAS Scale**		**N**		**Prevalence (%)**
No Anxiety		5 to 10		240		36.10
Moderate Anxiety		11 to 14		152		22.90
High Anxiety		15 to 18		143		21.50
Extreme Anxiety		19 to 25		130		19.50
	Mean	Median	Variance		Standard Deviation	
**MDAS Score**	13.73	13.00	30.35		5.51	

Table 1 also showed the different levels of dental concerns/fear about various triggers of dental anxiety among the study participants. The study participants had a fairly equal level of dental concern/fear about the sound or vibration of the drill. Up to 57% expressed a low level of concern about the numb feeling from dental treatment while 45.2% said they have a high level of concern about injection in the mouth. About 41% and 44% had a low concern about the sound or feel of scraping during teeth cleaning and cold air in the mouth respectively. The study participants had high levels of concerns (67%) for both root canal treatment and extractions. About 62% had high dental concerns for panic attacks during treatment.

The mean MDAS was 13.73 ± 5.51 (Table 1). The prevalence of dental anxiety among the respondents was 63.90%. The prevalence of extreme anxiety was 19.50% while high anxiety and moderate anxieties were 21.50% and 22.90% respectively (See details in Table 1 on how the MDAS scores were categorized). As shown in Table 2, all predictor variables were statistically significant (*p* < 0.001) except age, dental visit, and education (see Table 2 for details).

**Table 2:** One-way ANOVA: independent variables versus mean MDAS score

	F-value	p-value
Sex	10.02	0.002
Age group	0.38	0.822
Dental visit	2.36	0.166
Frequency of dental visit	7.22	1.198e-4
Household Income	2.62	0.042
Level of Education	0.96	0.399
Fear of sound or vibration of the drill	156.20	< 2.2e-16
Dislike the numb feeling	40.45	2.99e-15
Fear of injection in the mouth	92.74	< 2.2e-16
Fear of sound or feel of scraping during teeth cleaning	62.69	< 2.2e-16
Fear of cold air on the teeth	34.98	4.316e-14
Fear of root canal treatment	114.26	< 2.2e-16
Tooth removal	158.04	< 2.2e-16
Fear of being injured	112.39	< 2.2e-16
Fear of panic attacks	112.20	< 2.2e-16
Fear of feeling pain during treatment	136.78	< 2.2e-16
Concern of being embarrassed	43.79	3.41e-16
Dislike Smells in the dental office	72.48	< 2.2e-16
Fear of needing a lot of dental treatment	73.36	< 2.2e-16
Fear of cost of the dental treatment	6.53	1.69 e-3

### Multivariable analyses

#### Classical statistical model (multiple Linear regression)

As shown in Table 3, after careful consideration of all possible fitted models and their interaction terms for multiple linear regression analysis, the parsimonious model had 9 variables with Akaike Information Criteria (AIC) of 2376.4. The RMSE and R^2^ were 3.16 and 0.67, respectively.

**Table 3:** Summary output of the final (reduced) model from generalized linear model (Arranged in the order of variable importance based on the magnitude of their beta coefficient)

Final model: Akaike Information Criteria (AIC) = 2376.4			
	Categories	Estimate	P-value
Intercept		6.55	< 0.001
Fear of panic attacks	High	3.14	< 0.001
	Moderate	1.28	0.003
	Low	***Ref***	
Fear of feeling pain during treatment	High	2.76	< 0.001
	Moderate	0.88	0.09
	Low	***Ref***	
Fear of Sound or vibration of the drill	High	2.70	< 0.001
	Moderate	0.98	0.02
	Low	***Ref***	
Fear of root canal treatment	High	2.69	< 0.001
	Moderate	1.62	0.01
	Low	***Ref***	
Fear of injection in the mouth	High	1.91	< 0.001
	Moderate	0.42	0.37
	Low	***Ref***	
Fear of needing a lot of dental treatment	High	1.60	0.003
	Moderate	0.70	0.10
	Low	***Ref***	
Fear of being embarrassed	High	1.52	0.01
	Moderate	0.35	0.41
	Low	***Ref***	
Frequency of dental visit	Less than 3 months	-0.94	0.02
	3 months to less than 6 months	-1.17	0.01
	6 months to less than 12 months	-0.74	0.09
	12 months and above	***Ref***	
Fear of the sound or feel of scraping during teeth cleaning	High	0.70	0.12
	Moderate	0.82	0.04
	Low	***Ref***	

Dispersion parameter for gaussian family taken = 10.48

Null deviance: 13749.2 on 453 degrees of freedom

Residual deviance: 4546.4 on 434 degrees of freedom

Holding all other variables in the model constant, high and moderate fear of panic attacks during treatment were significantly associated with higher mean DA compared to low fear of panic attacks (β = 3.14, *p* < 0.01; β = 1.28, *p* < 0.01, respectively). High fear of feeling pain during treatment was associated with a higher mean DA compared to low fear (β = 2.70, *p* < 0.01). High and moderate fear of the sound or vibration of drills was associated with a higher mean DA score compared to having low fear (β = 2.70, *p* < 0.01; β = 0.98, *p* = 0.02, respectively). High and moderate fear of root canal treatment was associated with higher mean DA compared to low fear of root canal treatment (β = 2.69, *p* < 0.01; β = 1.62, *p* = 0.01). High fear of injection was associated with higher mean DA compared to low fear of injection (β = 1.91, *p* < 0.01). High fear of needing a lot of dental treatment was significantly associated with higher mean DA compared to low fear (β = 1.60, *p* < 0.01). High fear of being embarrassed was significantly associated with higher mean DA compared to low fear of being embarrassed (β = 1.52, *p* = 0.01). The frequency of dental visits of less than 3 months and 3 months to less than 6 months were associated with a lower mean DA score compared to dental visit frequency of 12 months and above (β = -0.94, *p* = 0.02; β = -1.17, *p* = 0.01). Moderate fear of the sound or feel of scraping during teeth cleaning was associated with a higher mean DA compared to low fear (β = 0.82, *p* = 0.04).

#### Machine learning model

After a comparison of the Lasso regression and XGBOOST model, the Lasso regression model was found to be the best-performing model with a mean RMSE of 0.617, R^2^ of 0.615, and MAE of 0.483 (Table 4). The details of the performance of the XGBOOST model can be found in Appendix VII. The calibration plot of the Lasso regression model showed a close calibration between the observed and the predicted means (Appendix III). The variable importance assessment found identified 28 predictors of dental anxiety in this population and these variables were ranked based on their permuted mean RMSE score (Table 5).

**Table 4:** **S**howing the performance of the LASSO regression model

Metrics	Mean	Median	SD	Min	Max
RMSE	0.617	0.597	0.040	0.585	0.672
R^2^	0.615	0.638	0.056	0.525	0.660
MAE	0.481	0.480	0.020	0.457	0.512

RMSE = Root mean square error; R^2^ = Coefficient of determination; MAE = Mean absolute error

**Table 5:** **S**howing variable importance based on permitted mean RMSE.

	Permuted mean RMSE*
High fear of feeling pain during treatment	0.093
High fear of sound or vibration of the drill	0.062
High fear of root canal treatment	0.061
High fear of panic attacks	0.052
High fear of injection in the mouth	0.043
High fear of needing a lot of dental treatment	0.026
High fear of being embarrassed	0.020
Moderate fear of root canal treatment	0.014
Moderate fear of panic attacks	0.012
High fear of being injured	0.012
Moderate fear of the sound or vibration of the drill	0.011
Frequency of dental visits (3 months to less than 6 months)	0.010
Frequency of dental visits less than 3 months	0.006
Moderate fear of sound or feel of scraping during teeth cleaning	0.005
High fear of cost of the dental treatment	0.003
High fear of cold air on the teeth	0.003
High dislike of smells in the dental office	0.003
Moderate fear of needing a lot of dental treatment	0.003
High fear of sound or feel of scraping during teeth cleaning	0.003
High fear of tooth removal	0.002
Moderate fear of concern of being embarrassed	0.002
Moderate fear for the numb feeling	0.002
Moderate fear of tooth removal	0.001
Frequency of dental visits (6 months but less than 12 months)	0.001
Moderate fear of injection in the mouth	0.001
Moderate dislike of smells in the dental office	0.001
Moderate fear of feeling pain during treatment	0.000
Sex (Male)	0.000
Moderate fear of cost of the dental treatment	0.000
High dislike the numb feeling	0.000
Moderate fear of being injured	-0.0004
Moderate fear of cold air on the teeth	-0.0005

*Higher permuted mean RMSE score means higher variable importance

#### Comparing variable importance of ML versus classical statistical model

As shown in Table 3, the classical statistical model had only 13 statistically significant variables and as such we selected the top 13 predictors of DA from the ML model variable importance estimation for comparison (Table 5). As shown in Table 6, both model types identified 12 similar variables (out of 13) as the most important predictors of dental anxiety in this study population. The ranking of the variable importance across the different model types varied however the top 5 predictors of DA were high fear of pain, panic attack, sound or vibration of drill, root canal, and injection in the mouth. The RMSE and R^2^ of the classical statistical model were 3.16 and 0.67, respectively versus the RMSE and R^2^ of the ML model which were 0.617 and 0.615, respectively (Table 6).

**Table 6:** Comparison of the 10 most important predictors of DA identified using ML model versus classical statistical model

ML model (Lasso regression) *	Classical statistical model (Multiple Linear regression) **
High fear of feeling pain during treatment	High fear of panic attack
High fear of the sound or vibration of the drill	High of feeling pain during treatment
High fear of root canal treatment	High fear of the sound or vibration of the drill
High fear of panic attacks	High fear of root canal treatment
High fear of injection in the mouth	High fear of injection in the mouth
High fear of needing a lot of dental treatment	Moderate fear of root canal treatment
High fear of being embarrassed	High fear of needing a lot of dental treatment
Moderate fear of root canal treatment	High fear of being embarrassed
Moderate fear of panic attacks	Moderate fear of panic attacks
High fear of being injured	Frequency of dental visits (3 months to less than 6 months)
Moderate fear of the sound or vibration of the drill	Moderate fear of the sound or feel of scraping during teeth cleaning
Frequency of dental visits (3 months to less than 6 months)	Moderate fear of the sound or vibration of the drill
Frequency of dental visits less than 3 months	Frequency of dental visits less than 3 months
**Comparison of performance of the ML model versus classical statistical model**	
RMSE = 0.617	RMSE = 3.16
R2 = 0.615	R2 = 0.67

* Ranking was based on the permuted mean RMSE.

** Ranking was based on the beta coefficient and statistical significance (*p* < 0.05)

Note: Lower RMSE equals better model performance.

## Discussion

Dental anxiety is a huge concern for dental professionals, public health specialists, and patients because of its association with poor oral health outcomes. Recent studies have shown a surge in the prevalence of dental anxiety [25, 26]. However, there are very few recent studies on young adults, especially in the United States of America [27, 28]. There are no existing studies that compared classical statistical models versus machine learning models in predicting and identifying the predictors of dental anxiety.

The high mean MDAS score found in this study is higher than the average MDAS score of 12.34 found in the Saatchi et al. study [29]. Comparable studies in the U.S. by Locker and Liddle (mean DAS = 7.8) and Kaako et al. study among university students (mean DAS = 9.2) have also shown lower scores even though they used DAS for measurement [13, 16]. It is crucial to highlight that the DAS (Corah’s Dental Anxiety Scale) comprises four items, yielding scores from 4 to 20. In contrast, the MDAS (Modified Dental Anxiety Scale) utilized in our study is a five-item measure, with score range of 5 to 25. Most of the study participants were females below age 25 and come from lower-income households. Almost all the participants had visited a dentist before and therefore have had some prior exposure to the clinical triggers of dental anxiety assessed in this study.

When compared to the ML model approach, the classical statistical model approach showed a much higher RMSE and slightly higher R^2^. This implies that our ML model performed better than the classical statistical model in predicting dental anxiety due to the higher error rate in the classical statistical model and bearing in mind that R^2^ is sensitive to the number of variables in the model and therefore not a very accurate measure of model performance. Also, our classical statistical model (multiple linear regression) revealed only 13 predictors of DA in this study population based on the beta coefficient and p-value. In contrast, our ML model (Lasso regression) identified 28 predictors of DA based on the permuted mean RMSE. This highlights the ability of machine learning to model complex interactions between variables and identify a wider range of predictors beyond the classical model.

Interestingly, our study showed a very comparable performance between the classical statistical modelling approach and the machine learning approach in terms of variable selection. When we compared the 13 predictors of DA from the classical statistical model to the top 13 predictor variables from the machine learning model, both models identified 12 similar predictors of dental anxiety. The predictors include high fear of feeling pain during treatment, high fear of the sound or vibration of the drill, high fear of root canal treatment, high fear of panic attacks, high fear of injection in the mouth, high fear of needing a lot of dental treatment, high fear of being embarrassed, moderate fear of root canal treatment moderate fear of panic attacks, moderate fear of sound or vibration of the drill, frequency of dental visit (3 months to less than 6 months) and frequency of dental visit (less than 3 months).

High fear of pain during treatment and fear of injection in the mouth were associated with higher dental anxiety. More than half of the participants rated their level of fear of pain during treatment as high while more than three-quarters of the participants indicated moderate to high levels of fear of injection. Our findings align with the studies from Georgelin-Gurgel et al. [30] that found an association between higher levels of DA and fear of intraoral injection. Individuals who had high fear of the sound or vibration of drills had a 2.70 higher mean DA score compared to those who had low low fear of the sound or vibration of drills. This agrees with the Cohen et al.28 study that found a significant relationship between DA and the sound/vibration of drills.

High fear of root canal treatment was associated with a 2.6 higher mean DA score compared to low fear of root canal treatment with more than about two-thirds of the participants indicating high level of fear about root canal treatment which aligns with similar findings by Alghofaily et al. [31].

Individuals with high fear of panic attacks during treatment had a 3.22 higher mean DA score compared to those who had low panic attacks during treatment. Also, participants with high fear of being embarrassed had a 1.5 higher mean DA compared to those who had low fear of being embarrassed. A high level of concern about needing a lot of dental treatment was significantly associated with 1.84 higher mean DA compared to a low level of concern about needing a lot of dental treatment. The public health relevance of these findings is that if an individual feels they are going to be embarrassed or get diagnosed with more dental issues, they become more anxious and avoid routine dental care visits altogether.

Individuals who visited the dentist more frequently were significantly less likely to have dental anxiety. Individuals who have visited a dentist in the past 6 months had at least a 1.18 lower average DA score compared to those who have not visited the dentist in the past 12 months. This finding conforms with the study by Doerr et al. study where those who did not go for a checkup at least once a year were found to be more dentally anxious than subjects receiving more frequent dental care [27]. This implies that frequent visits to the dentist could help decrease dental anxiety due to continuous exposure to dental anxiety stimuli thereby improving the patient’s self-efficacy. Inversely, high dental anxiety can be said to have caused the low frequency of visits within this study population possibly due to previous personal experience or experiences of a family member, friends, or colleagues. Our study found no association between dental anxiety and the average household income, level of education, and sex. Both Our Machine Learning (ML) and the classical statistical approach identified set of variables as predictors for dental anxiety. These variables hold the potential to form the basis for developing a web application tailored to aid the diagnosis of dental anxiety and the customization of patient-specific interventions. It is important to highlight that our findings were derived from a sample of 454 students, which differs greatly from the initially calculated sample size of 1062 students and may have limited external validity.

The limitations of this study include the lack of generalizability to other populations due to the differences in population characteristics. The data obtained from WIU might not be representative of other universities in America. The study participants might not have given adequate and accurate information regarding the level of dental anxiety since its measurement is subjective. Reliability testing of the modified DCA questionnaire was not conducted, therefore caution should be applied when using the modified DCA questionnaires outside this study population. Similarly, it was not feasible to determine the reasons behind non-responses from certain study participants or elucidate factors implicated in the low response rates. This limitation may have significant implications for the strength of our study’s conclusions. However, a smaller sample size may affect the statistical power of our analysis, which may underestimate the actual effect or relationships present in this population.

Also, due to the sampling method and sensitivity of the topic, the true cases might have been missed out. Other than comparing the variable selection, there are no existing objective metrics for comparing classical statistical versus machine learning models.

In future studies, a more diverse and larger sample size will be considered to enhance the strength, reliability and applicability of our study.

## Conclusion

There is a high burden of dental anxiety within this study population and continues to constitute a serious dental public health issue because those impacted are known to avoid dental visits. More frequent exposure to the clinic environment through routine visits plays a huge role in reducing the burden of dental anxiety, especially in young adults. This study contributes to reduce the knowledge gap about the impact of clinical exposure variables on dental anxiety and the role of machine learningin the prediction of dental anxiety. Behavioral theory (such as motivational interviewing) based public health interventions that are geared towards eliminating the individual clinical exposure triggers of dental anxiety are recommended.

### Electronic supplementary material

Below is the link to the electronic supplementary material.

Supplementary Material 1

## Data Availability

The data that support the findings of this study are available on request from the corresponding author.
