# Supplementary material for: Predicting dental anxiety in young adults: classical statistical modelling approach versus machine learning approach
Source: BMC Oral Health. 2024 Mar 9;24:313. doi: 10.1186/s12903-024-04012-3 (PMC10924339; doi:10.1186/s12903-024-04012-3)

**Appendix I**: Showing bivariate relationship between MDAS score and each of the explanatory variable.


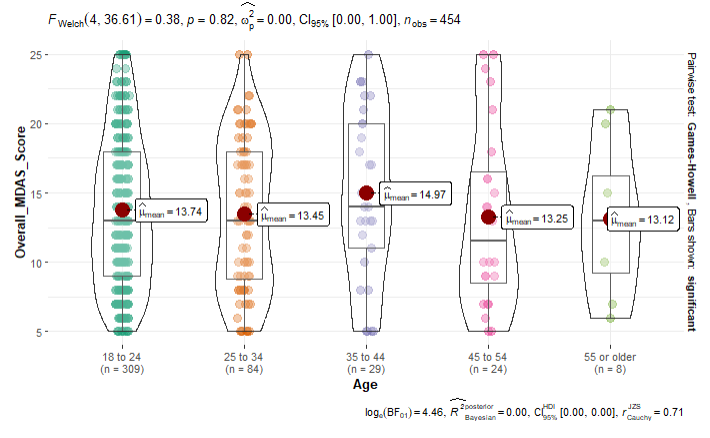


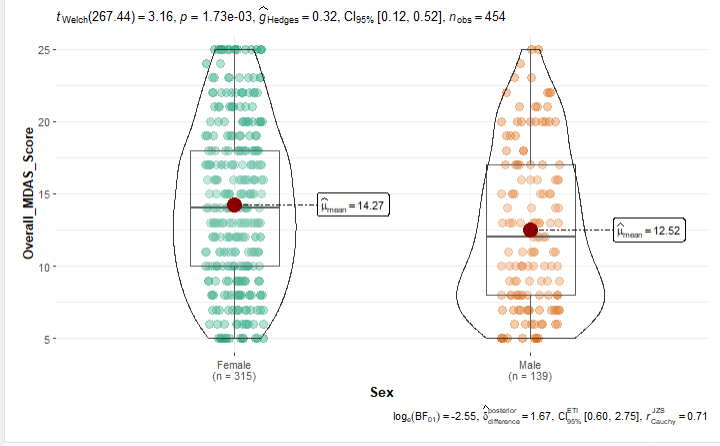


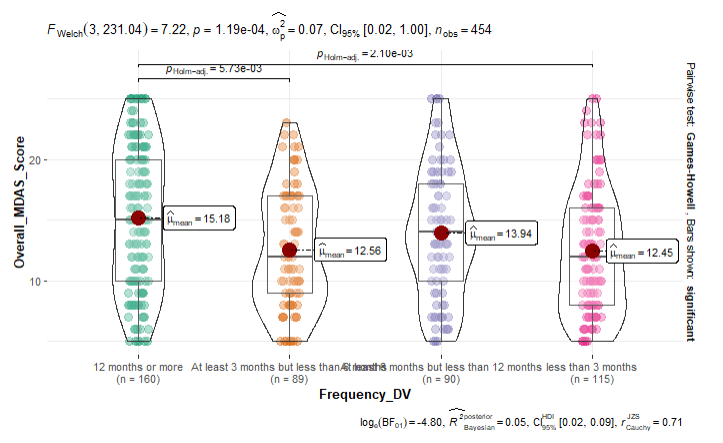

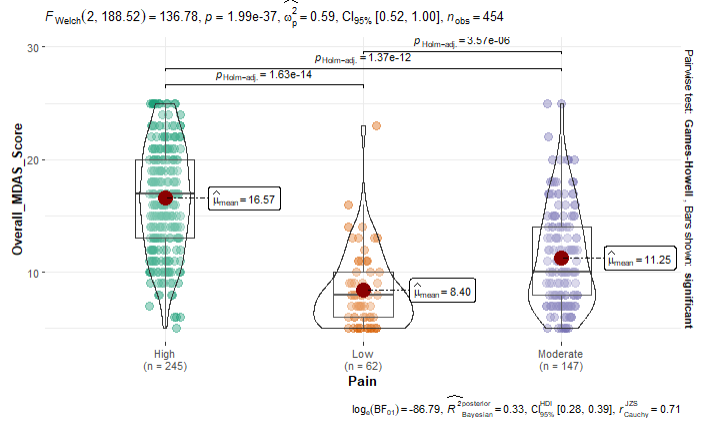


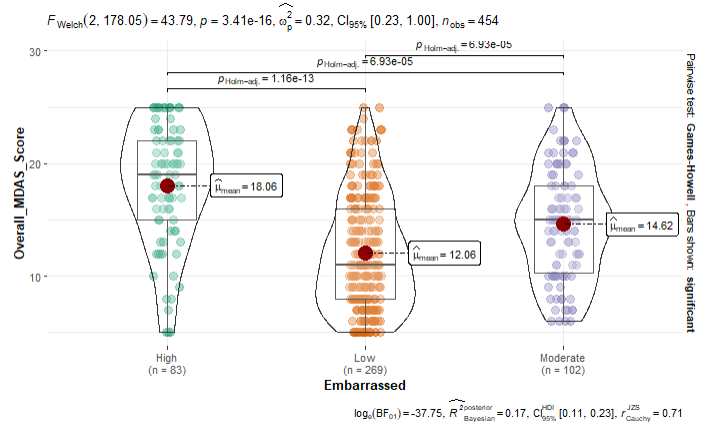

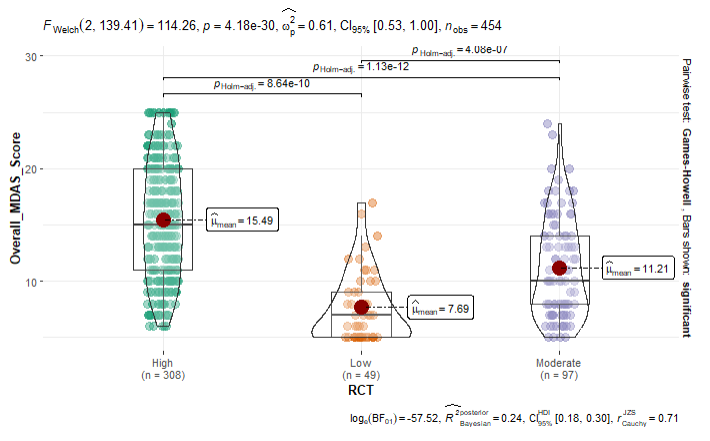


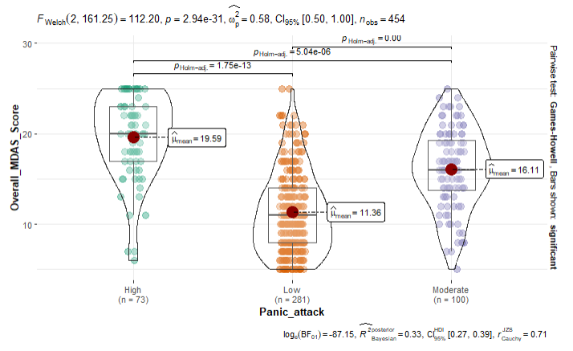


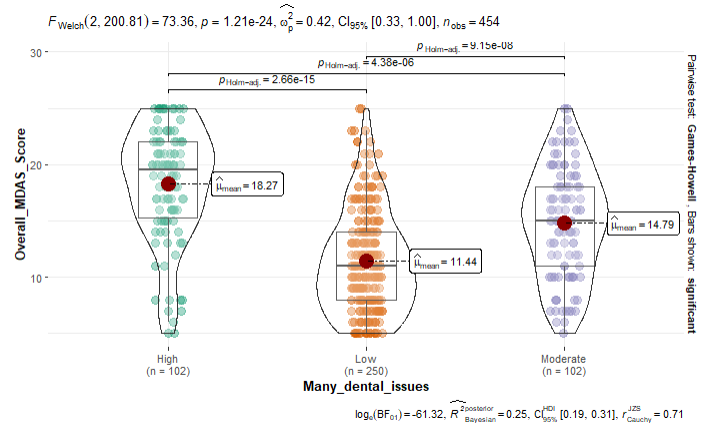


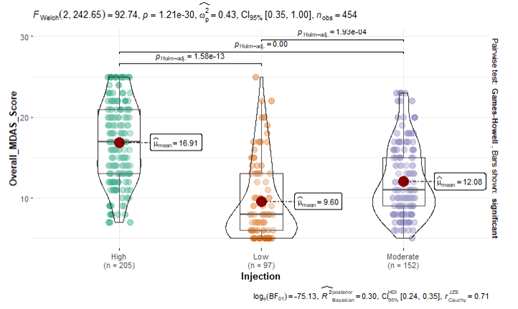


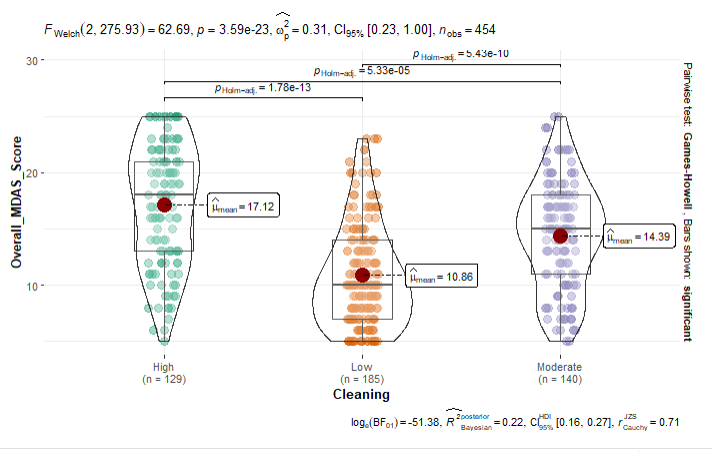


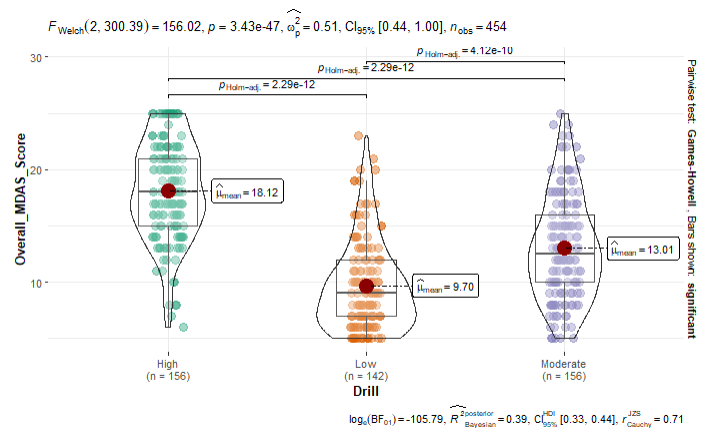


**
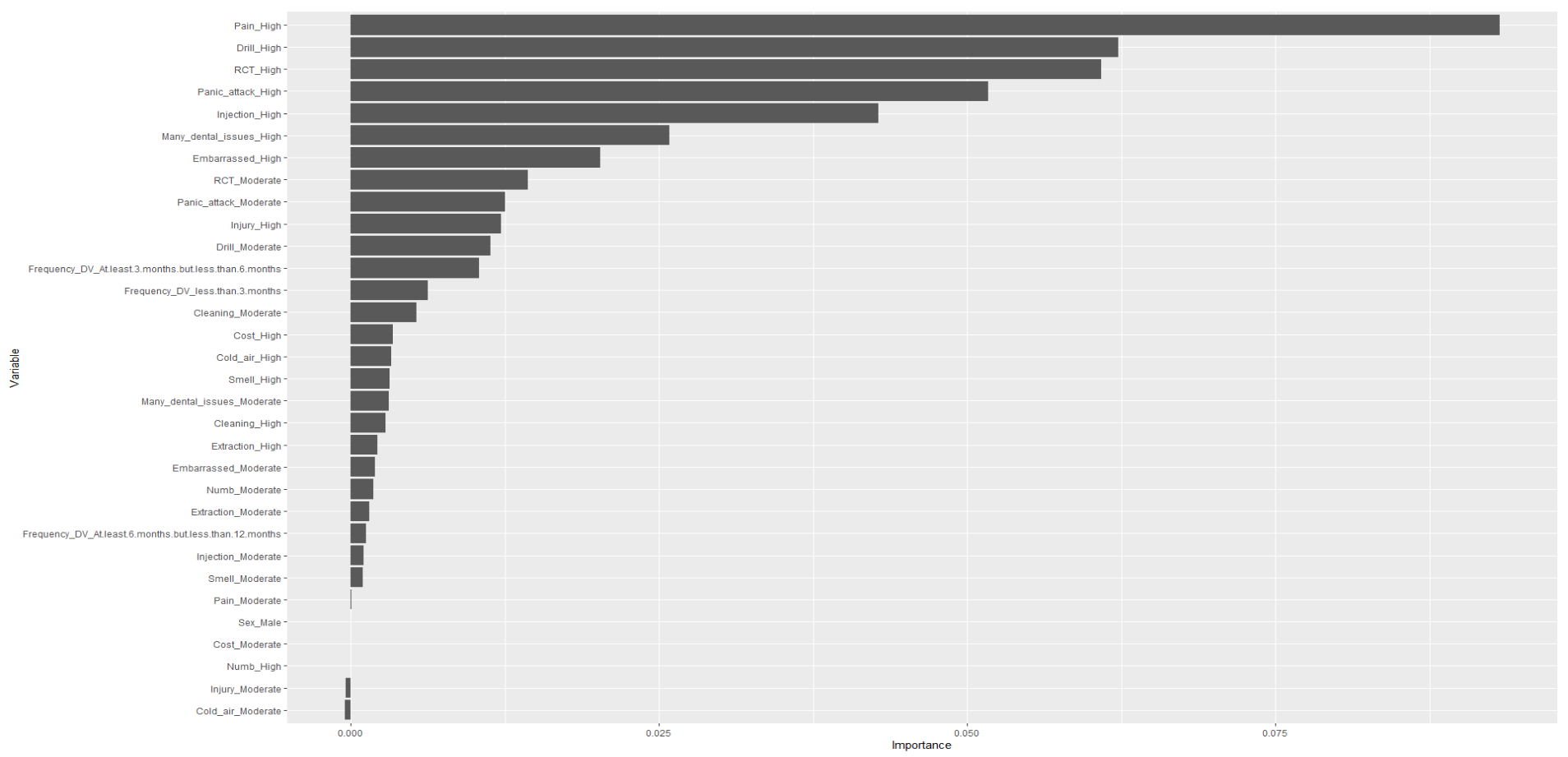
Appendix II.** Chart showing variable importance of the variables in the Lasso model.


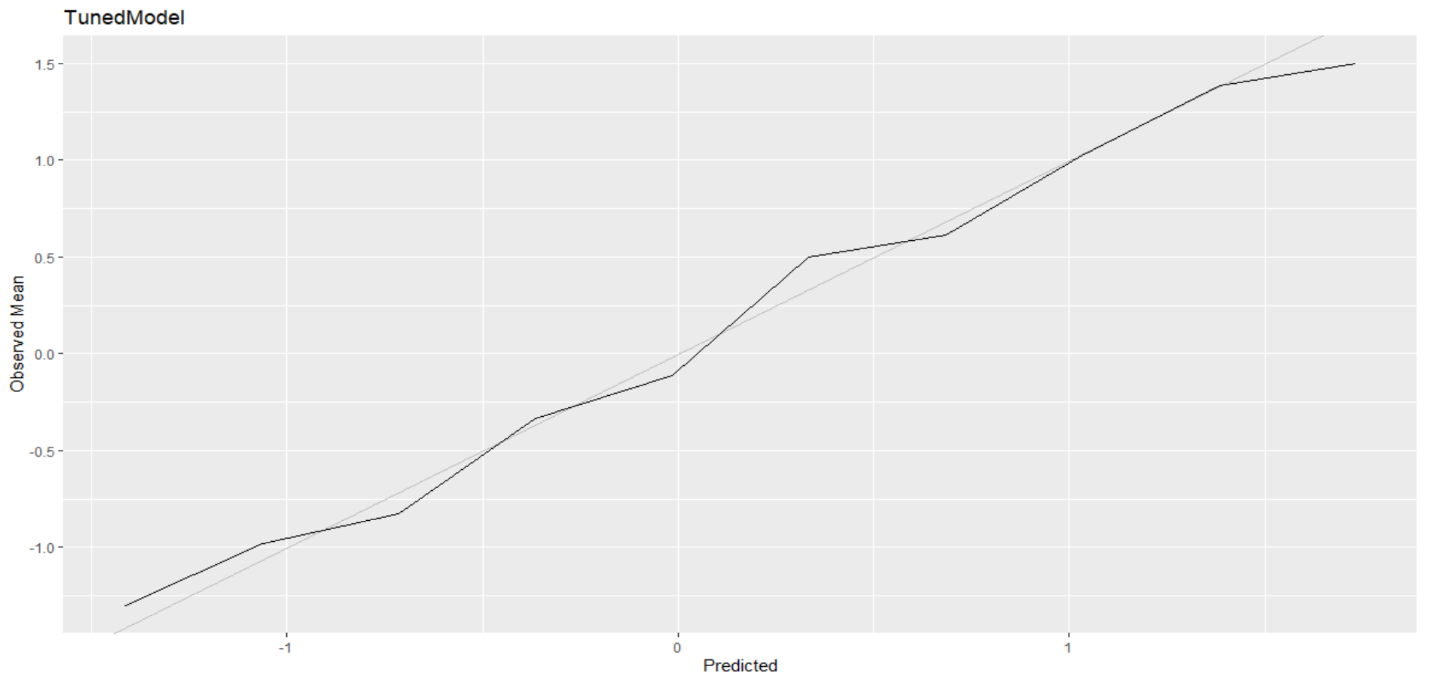
**Appendix III.** Calibration plot showing the relationship between the observed means and predicted means.


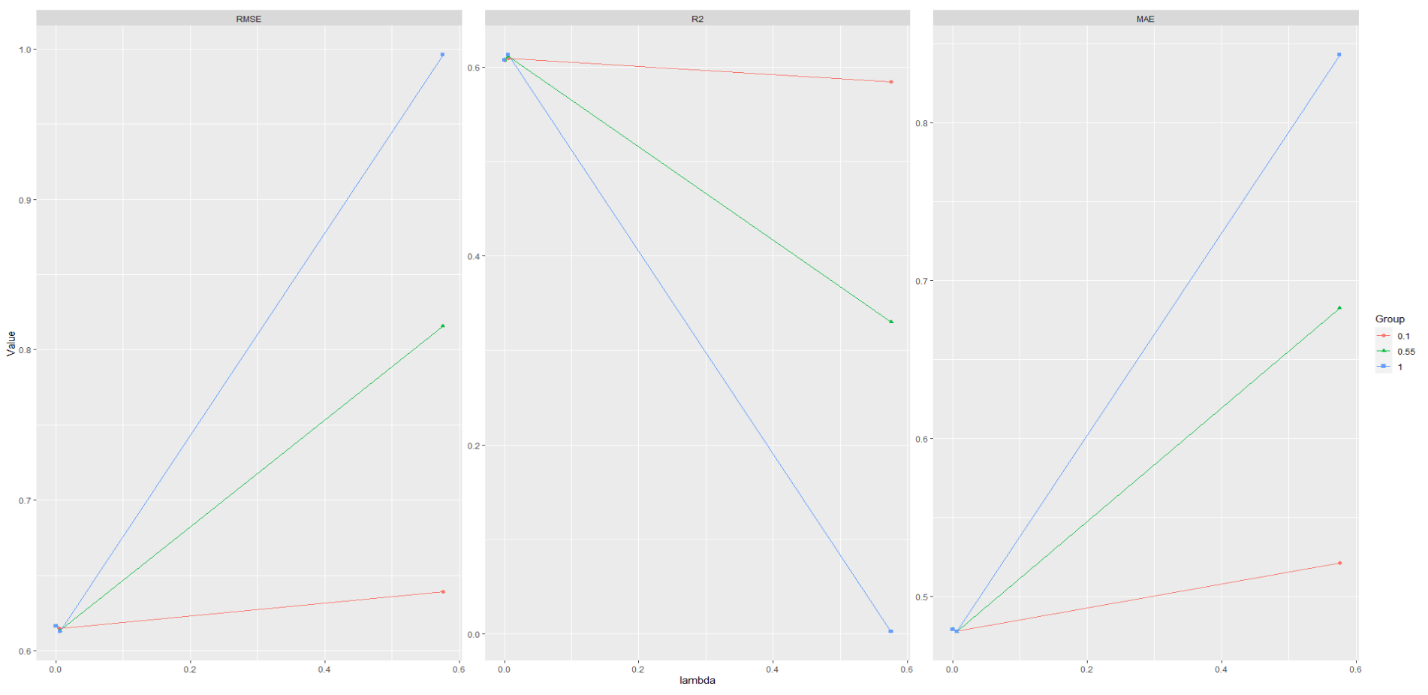
**Appendix IV.** Partial dependence plot showing the relationship between penalty function (Lambda) and the performance parameters (Lasso model)


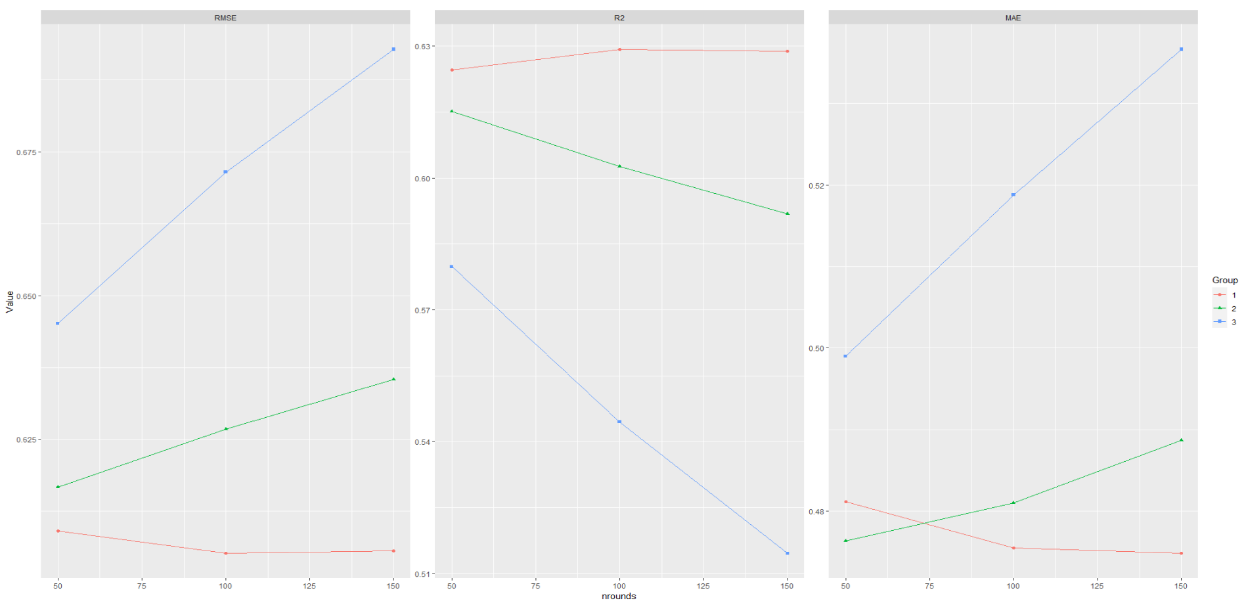
**Appendix V.** Partial dependence plot showing the relationship between penalty function (Lambda) and the performance perimeter. (XG-BOOST)

**Appendix VI.** Chart showing variable importance (XG-BOOST)


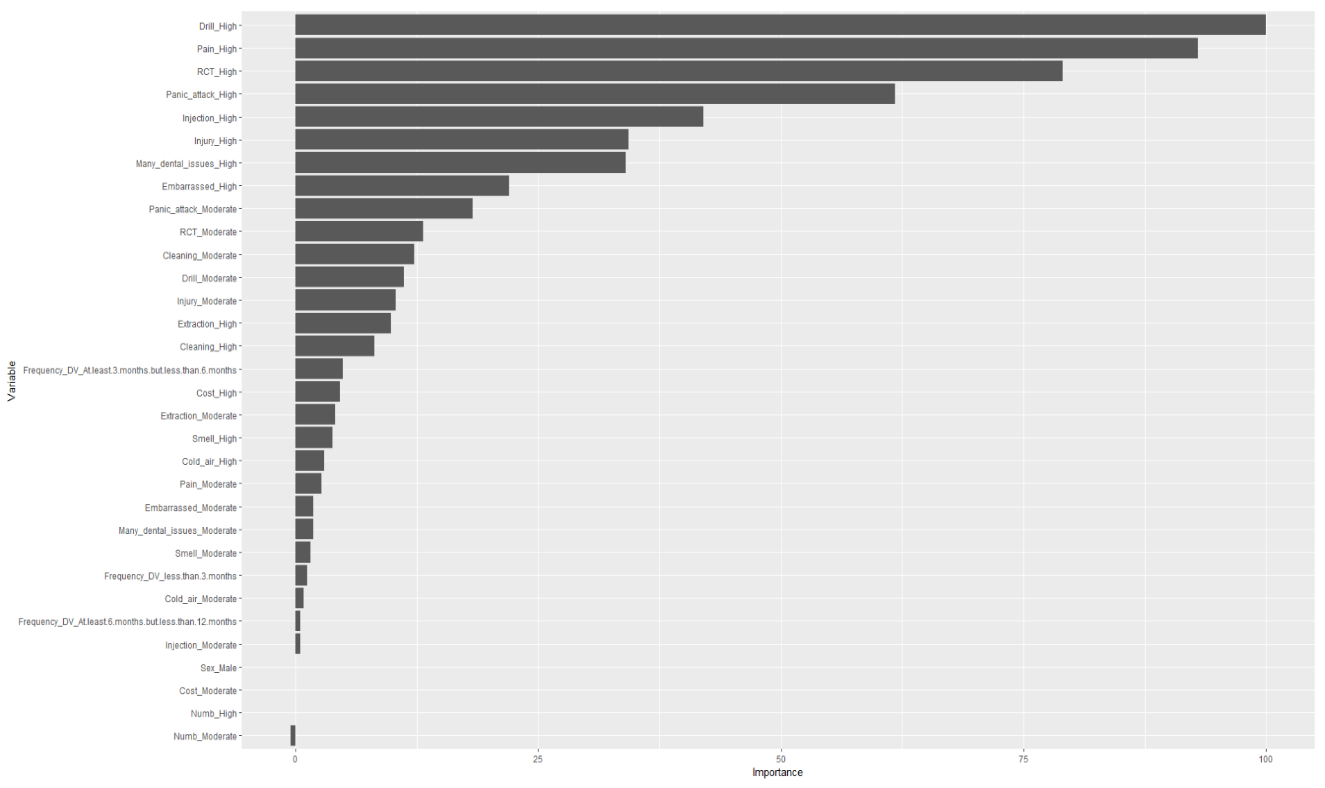


**Appendix VII. S**howing the performance of the XG-BOOST regression model.

| Metrics | Mean | Median | SD | Min | Max |
| --- | --- | --- | --- | --- | --- |
| RMSE | 0.618 | 0.622 | 0.035 | 0.585 | 0.667 |
| R^2^ | 0.615 | 0.630 | 0.048 | 0.532 | 0.654 |
| MAE | 0.483 | 0.476 | 0.022 | 0.455 | 0.507 |
| RMSE = Root mean square error; R^2^ = Coefficient of determination; MAE = Mean absolute error | | | | | |

**Appendix VIII.** Calibration plot showing the relationship between the observed mean and predicted mean (XG_BOOST).


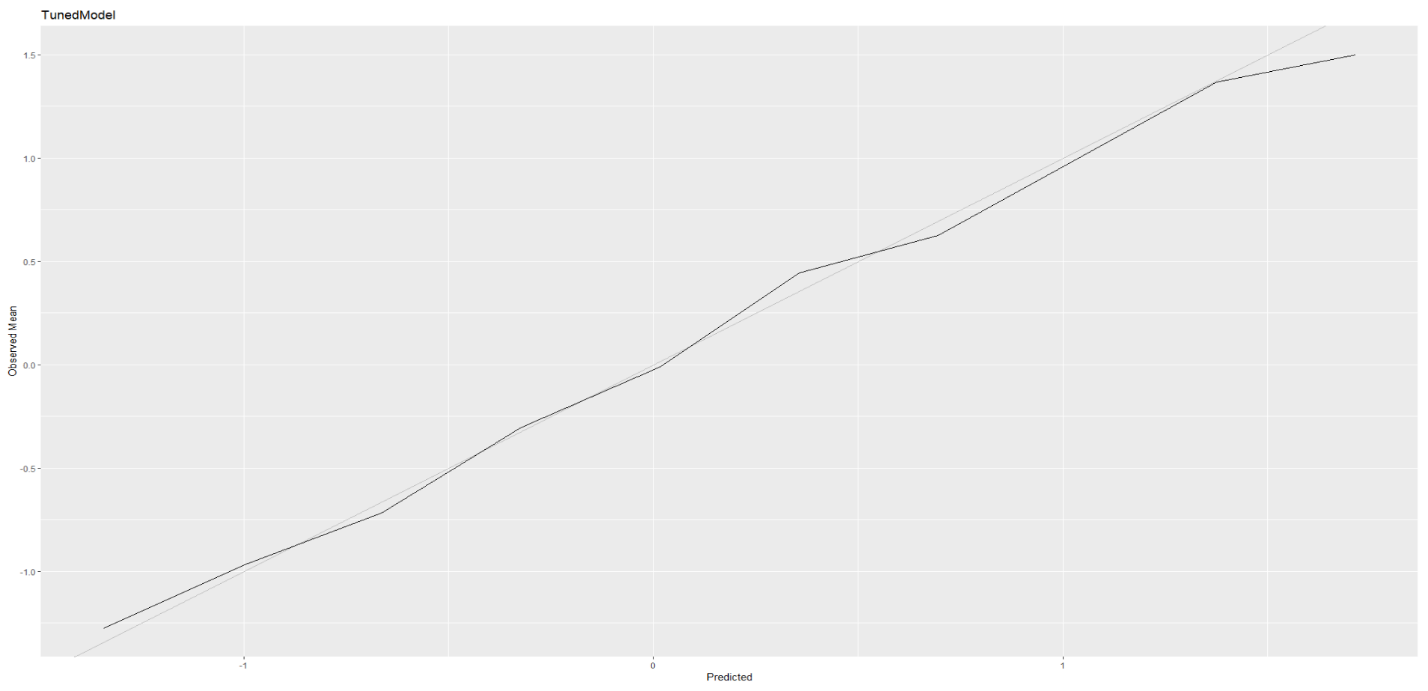

Supplement: Supplementary file 1 — Supplementary Material 1 [file 12903_2024_4012_MOESM1_ESM.docx]
